# Improving clinical efficacy of adeno associated vectors by rational capsid bioengineering

**DOI:** 10.1186/s12929-014-0103-1

**Published:** 2014-11-26

**Authors:** Dwaipayan Sen

**Affiliations:** School of Biosciences and Technology, Vellore Institute of Technology (VIT) University, Vellore, 632014 Tamil Nadu India

**Keywords:** Adeno associated virus, Capsid bioengineering, AAV, Site directed mutagenesis

## Abstract

Adeno associated vectors (AAV) have shown considerable promise to treat various genetic disorders in both preclinical and clinical settings mainly because of its safety profile. However, efficient use of AAV to deliver genes in immune-competent sites like muscles and liver requires very high doses which are associated with concomitant cellular immune response against the viral capsids leading to destruction of the transduced cells. Coupled with that, there are enough evidences that at high doses, AAV particles are subjected to increased cellular phosphorylation/uniquitination leading to proteasome mediated degradation and loss of the viral particles. The presence of preexisting immunity against AAV further adds on to the problem which is acting as a major roadblock to efficiently use it as a gene therapy vector in the clinics. To overcome this, rational bioengineering of AAV capsid becomes a prime tool by which specific amino acid residue(s) can be suitably modified/replaced by compatible residue(s) to create vectors having lower host immune response and higher intracellular trafficking rate. This article reviews the various aspects of rationally designing AAV capsids like by site-directed mutagenesis, directed evolution and combinatorial libraries which can create vectors having not only immune evasive property but also enhanced gene expression and transduction capability. One or more combinations of these strategies have strong potential to create novel vectors which will have suitable clinical efficiency even at a low dose.

## Introduction

### AAV Biology

Adeno-associated virus (AAV) is a non-pathogenic parvovirus that has been widely used as a vector of choice for gene therapy. Although the virus has been detected in many different tissues of several animal species [1] it has not been associated with any disease [2,3]. Coupled with its ability to transduce both dividing and non-dividing cells, and low immunogenicity, AAV makes an exciting candidate for a gene therapy vector. Its genome is composed of a 4.7-kb single-stranded DNA packaged into a non-enveloped, icosahedral capsid [4]. The single stranded genome encodes for three open reading frames (ORF) placed in between two inverted terminal repeats (ITR), which helps in packaging by acting as the origin of replication. Viral replication, transcription, assembly and site specific integration is regulated by the four non-structural proteins (Rep78, Rep68, Rep52, and Rep40) [5] encoded by the rep ORF. A 60-mer capsid is formed by the assembly of three structural proteins (VP1, VP2, and VP3) encoded by the cap ORF. The capsid proteins responsible for viral entry into the cells recognizes specific receptors on the cell surface leading to receptor mediated endocytosis from clathrin-coated pits [6]. In recombinant AAV, the gene of interest is included between the ITRs while the rep and cap ORFs are supplied in trans. Thus current recombinant AAV (rAAV) vectors persist primarily as extra-chromosomal elements [7,8]. AAV has been used for *in vivo* gene transfer to various target tissues like muscle, liver, retina, lung or the brain. Despite the reported success it is becoming increasingly clear that humoral and cell mediated immune response against the vector is a major impending factor towards the efficacy of gene therapy [9]. Preexisting neutralizing antibodies and antigen specific T cells recognizing AAV capsid proteins against AAV capsids has been shown to negatively impact the vector transduction and sustained gene expression by immune mediated clearance of the transduced cells expressing the capsid proteins [10,11].

### AAV and clinical trials- the problem of immune mediated clearance of AAV vectors

AAV has been used in several clinical trials for both inherited and non-inherited diseases with considerable success (Table 1). In the phase-I dose escalation trial for Leber’s congenital amaurosis (LCA), all 12 patients who received a subretinal injection of AAV2 encoding a protein required for isoamerohydrolase activity of retinal pigment epithelium demonstrated improved vision [12] with no significant immunological or toxic adverse events [12,13]. As another example, AAV has also been used to treat a chronic neurodegenerative disorder called Parkinson’s disease. A study in which [14,15] 12 patients with advanced Parkinson’s disease, AAV vector carrying a gene encoding glutamic acid decarboxylase was injected into the subthalamic nucleus on one side. Following injection, motor activity on the treated side was improved significantly relative to the untreated side which was persistent for atleast one year. Most importantly there were no adverse affects attributable to gene therapy even at the highest dose.

**Table 1:** **AAV in clinical trials**

**AAV serotype**	**Disease**	**Administration route**	**Trial Number (** **www.clinicaltrial.gov** **)**
rh.10	Late Infantile Neuronal Ceroid Lipofuscinosis	Intracranial	NCT01161576
1	Pompe disease	Intradiaphragmatic	NCT00976352
2	Leber Congenital Amaurosis	Subretinal	NCT00643747; NCT00516477; NCT00999609; NCT00749957
2	Retinal disease (MERTK mutation)	Subretinal	NCT01482195
2.8	Hemophilia B	Intramuscular, hepatic, intravenous	NCT00515710; NCT01687608; NCT00979238; NCT01620801
2	Idiopathic Parkinson’s Disease	Intracranial	NCT00985517
1,2	Alpha-1 Antitrypsin Deficiency	Intramuscular	NCT00430768; NCT01054339; NCT00377416
1	Lipoprotein Lipase Deficiency	Intramuscular	NCT00891306; NCT01109498
2.5	Duchenne Muscular Dystrophy	Intramuscular	NCT00428935
2	Cystic Fibrosis	Intranasal, endobronchial	NCT00004533
2	Rheumatoid arthritis	Intraartiular	NCT00617032, NCT00126724
2	Age-related macular degeneration	Intravitreal	NCT01024998
1,6	Severe heart failure	Intracoronary	NCT00454818; NCT00534703

In contrast to these clinical studies, which targeted immune privileged sites, AAV has limited success when it came to treat monogenic diseases like haemophila B and lipoprotein lipase (LPL) deficiency following intravenous, intrahepatic or intramuscular administration (Table 2). For example in the first clinical trial for hemophilia B conducted by Katherine High’s group [11] there was strong-cell mediated immune response against the AAV capsid antigens in the high dose recipient subject which lead to destruction of the AAV2 transduced hepatocytes resulting in only transient therapeutic expression of Factor (F). IX (2 months). There was also a very steep increase in the neutralizing antibody titer against the capsid following vector administration [11]. In the recent hemophilia B clinical trial using self complementary AAV8 vector, [16] a similar problem was encountered in the high vector dose group. There was an increase in liver transaminases with concomitant drop in the circulating Factor IX levels 8 weeks post vector administration (Table 2). These findings were found to be because of capsid specific T-cell responses which lead to the loss of the transduced hepatocytes. Thus, overall the theme of dose dependent immune response against the AAV capsid is still a persisting and real problem [17].

**Table 2:** **Adverse immune response against AAV in haemophilia B clinical trials**

**Vector**	**No of subjects**	**Vector dose**	**Adverse Effect**	**Immune response**	**Reference**
AAV2	2	2X10^12 (high dose)	Liver toxicity based on elevated AST/ALT levels beginning 4 weeks post vector infusion with concomitant decline of circulating h.FIX to the baseline (<0.1%) by 8 weeks	CD8+ T cell response against AAV capsid as well as preexisting neutralizing antibody against AAV2 capsid prevented long term expression	[11]
AAV2	4	4X10^11 (low dose)	No increase in circulating hF.IX from the baseline (<0.1%), increased transaminases only in one subject having the lowest pretreatment NAb	CD8+ T cell response against AAV capsid as well as prexisting neutralizing antibody against AAV2 capsid prevented hF.IX expression	[11]
AAV8	1	2X10^12 (high dose)	Liver toxicity based on elevated AST/ALT levels beginning 8 weeks post vector infusion with concomitant decline of circulating hF.IX levels	CD8+ T cell response against AAV capsid leading to destruction of the transduced hepatocytes	[16]

## Review

### Strategies to avoid immune response against AAV capsid

#### Transient immune-suppression

One of the major barriers to successful gene delivery with AAV vectors is the humoral immunity to wild type vectors. Humans are natural carriers of AAV genome. Neutralizing antibodies (NAb) to AAV (AAV1 and 2) in humans was first reported in the early 60s and 70s [18,19]. Recently, more than 100 natural AAV variants have been isolated from human and non-human primates tissue specimens [1,20,21]. AAV2, which is the most widely used and characterized serotype has a seroprevalence of almost 30-60% in samples from 10 countries and 4 continents (America, Europe, Africa, and Australia) [22]. In the naïve host, humoral immune responses are elicited upon AAV vector application. Transient immune-suppression is one of the ways that has been considered to circumvent this humoral response against AAV. Use of clinically approved immunosuppressive drugs like rituximab and cyclosporine in rhesus monkey which were systemically injected with AAV vector resulted in elimination of anti-F.IX NAb with restoration of plasma F.IX transgene product detection. When this finding was extended to humans, rituximab reduced neutralizing antibodies to AAV2 and 5 significantly in ~30% of subjects [23]. However immune-supression has its own disadvantage also. Manning and his group demonstrated that the use of antibodies and or small molecule inhibitors against CD40 was successful in vector readiministration, which was however dented following the second administration due to development of neutralizing antibodies (without immune-suppression) [24]. In another study, Jiang *et al.,* tried to transiently suppress the immune system to inhibit AAV capsid specific T cell response against transduced hepatocytes expressing F.IX transgene in rhesus macaques [25]. But no effect was found on the expansion of memory T cells in any of the animals. Also one of the three animals who received immune-suppression unexpectedly developed strong anti-AAV antibody response. Furthermore in this study neutralizing antibody titres increased dramatically upon withdrawal of the immune-supression therapy after 6 weeks indicating that the tolerogenic properties of AAV can be altered after prolonged immunosuppressive treatment.

To summarize, immune-suppression can be advantageous as it represses the body’s immune response long enough for the AAV capsid proteins to be not recognized by our defense mechanism thereby preventing NAb formation and allowing readministration of the vector. However this strategy will not be useful to circumvent preexisting NAb against AAV capsid. Thus, alternate strategies like rational capsid modifications must be looked into to evade these neutralizing antibodies.

#### Rational design of AAV variants by site-directed mutagenesis

The ubiquitin–proteasome pathway has been shown to play an essential role in AAV intracellular trafficking [26,27] and this pathway has been shown to be modulated by epidermal growth factor receptor protein tyrosine kinase (EGFR-PTK) signaling [28]. In this study, the authors found that inhibiting the EGFR-PTK signaling enhances the efficiency of AAV transduction by efficient second strand synthesis as well as increased viral trafficking from the cytoplasm to the nucleus. The same group later showed that EGFR-PTK is able to phorphorylate tyrosine residues on AAV capsids *invitro.* Extending this finding *invivo* the authors were able to elucidate a negative effect of tyrosine phosphorylation on viral intracellular trafficking and transgene expression [29]. Thus based on these findings it was hypothesized by the authors that phosphorylation of tyrosine residues on AAV capsid mediated by EGFR-PTK serves as a signaling for uniquitination of the capsid leading to proteasomal degradation in the cytoplasm before the viral particles can enter into the nucleus. Thus the authors carried out site directed mutagenesis of surface exposed tyrosines (tyrosine(Y) to phenylalanine (F)) on the AAV capsid (Y252, Y272, Y444, Y500, Y700, Y704, and Y730) and showed increased *invitro* (~10 fold) and *invivo* (hepatic, ~30 fold) transduction efficiency of the novel vectors.

Following this finding, tyrosine mutant AAV vectors were used in other target sites like retina [30], skeletal muscles [31], human hematopoietic stem cells (HSCs) [32], fibroblasts and mesenchymal stem cells [33] where it showed efficient transduction as compared to the wild type vectors. For example AAV 2-Y444F and Y730F, mutant Y733F in AAV-8, and mutant Y446F in AAV-9 demonstrated enhanced transduction efficiency in the retinal ganglion cell layer after intravitreal injection [34]. AAV6 is reported to be the most efficient vector for transducing muscles with ~500 fold higher efficiency than AAV2 vector [35]. In one study, the authors demonstrated high efficiency transduction in muscles using the tyrosine mutant AAV6-Y445F and AAV6-Y731F compared to WT-AAV6 (6–8 fold). More recently a novel double tyrosine mutant of AAV6 (Y705 + 731 F) demonstrated high-efficiency transduction of HSCs as well as expression of the β-globin gene in erythroid progenitor cells for the potential gene therapy of human hemoglobinopathies such as β-thalassemia and sickle cell disease.

**Figure 1:**
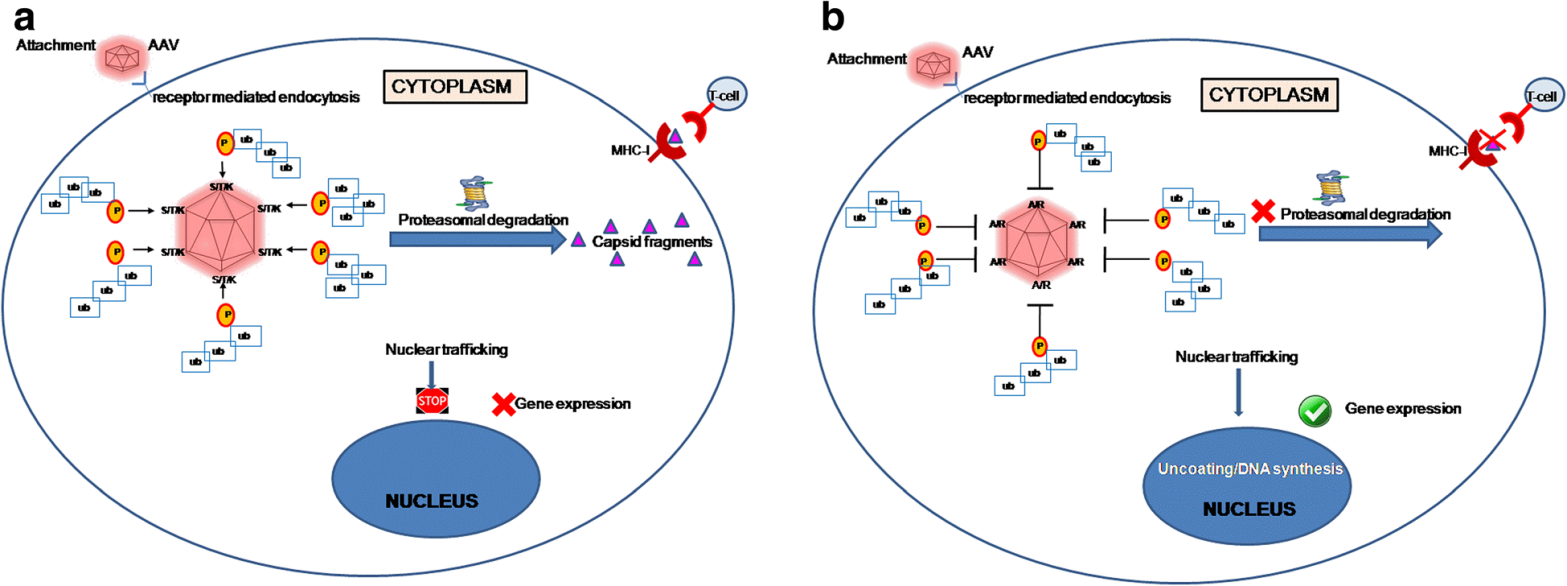
**Targeted mutation of S/T/K residues on AAV capsid.** Following cellular internalization of AAV by receptor-mediated endocytosis, it travels through the cytosol, undergoes acidification in the endosomes before getting released. Post endosomal escape, AAV undergoes nuclear trafficking, where uncoating of the viral capsid takes place resulting in release of its genome and induction of gene expression **(a)** S/T/K residues are potential sites for phosphorylation and subsequent poly-ubiquitination which serves as a cue for proteasomal degradation of capsid proteins. This prevents trafficking of the vectors into the nucleus to express its transgene leading to low gene expression. Also, the proteasomally degraded capsid fragments can be presented by the MHC-Class I molecules on the cell surface for CD8 + T-cell recognition. This leads to immune response thus destroying the transduced cells and further reducing persistent transgene expression. **(b)** Point mutations, S/T to A and K to R, prevents/reduces phosphorylation sites on the capsid. This leads to reduced ubiquitination and proteosomal degradation allowing more number of intact vectors to enter nucleus and express the transgene. Preventing/lowering the overall capsid degradation also reduces antigen presentation to T cells resulting in lower host immune response against the vectors. **ub**- ubiquitination, **p**- phophorylation.

Apart from tyrosines, serines (S), threonines(T) and lysines(K) are also potential sites for phosphorylation and or ubiquitination on the AAV capsids and traditionally mutating them could augment AAV transduction efficiency. It has been shown earlier that targeted inhibition of the serine/threonine kinase phosphorylation of a cellular protein FK506-binding protein (FKBP52), improved AAV mediated gene transfer by 30-fold compared to ~5 fold increase seen with inhibition of tyrosine kinases alone [36]. It is also known that lysine residues are direct targets for host cell ubiquitination [37] and therefore modifying them is likely to be reduce vector ubiquitination and subsequent proteasome mediated degradation. The degraded capsid proteins could also be presented to the T-cells via major histocompatibility complex (MHC)-class I leading to destruction of the transduced cells (Figure 1).

Going by this logic, several studies by our laboratory at Christian Medical College, Vellore, India, had generated S/T/K mutants on AAV1, 2, 5 and 8 which has proven to be more efficient in hepatic gene delivery as compared to their wild type counterparts [38-40]. S/T residues were mutated to alanine (A), while K were mutated to arginine (R). The residues chosen for mutation were selected based on structural predictions on their presence in and around phosphodegrons (phosporylation sites) on the AAV capsid structure as well as residues which lie in the antigenic epitopes that will create vectors which not only will be efficient in terms of higher transduction and gene expression, but also will have reduced neutralizing antibody response against the viral capsids allowing for persistent transgene expression. Indeed it was found that several vectors like AAV2-S489A, S662A, T251A, K544R, AAV5-S652A, AAV1-S669A, AAV8-K137R, S671A to demonstrate several fold increase in transgene expression (enhanced green fluorescence protein, luciferase or human F.IX) in murine liver as compared to their WT vectors [38-40]. Also, the AAV8-K137R mutant vector showed reduced hepatic inflammatory response as well as reduced neutralizing antibody response (2 fold) in comparison to the wild type vector.

In summary, rational point mutations on AAV capsids have shown considerable promise and this field is still wide open to explore especially since we have access to the 3 dimensional (3D) structures of several clinically important AAV serotypes [41-49]. Using the 3D models, exact prediction of phosphorylation/ubiquitination and antigen recognition epitopes are possible which will give scientists more confidence to modify those regions. For example, it is important to understand that phosphorylation of the viral capsid serves as a trigger for uncoating and release of viral genome inside the host cell. Thus phosphorylation sites cannot be replaced randomly and have to be mutated strategically. Knowledge of the 3D structure enables us to choose the phosphorylation sites to mutate safely within the phosphodegrons as they are the ones that are used by host as a signal for clearance of the virus. These residues are thus expected to have minimum effect on the capsid uncoating processes, essential for gene expression inside host cells. Also, to preserve capsid geometry, only those residues that lie outside the interaction interfaces in the phosphodegron can be selected for mutagenesis. Knowledge of the 3D structures also allows us to avoid mutating any residues which falls in the capsid interaction domains or receptor binding domains thus preserving their infectivity and tropism. Recently, with help of the 3D structural information, *Tenney et al.,* [50] could define the amino acids responsible for AAV8’s high hepatic transduction efficiency. In this study, the authors created chimeric AAV2 vectors (containing amino acid residues from the AAV8 capsid variable regions at the two fold symmetry axis) that were found to transduce murine liver cells nearly as well as AAV8. More importantly, knowledge of the antibody binding domain (s) and or T- cell recognition epitope (s) from 3D structural analysis coupled with wet laboratory experiments will enable us to save time and enforce informed judgment on choosing the appropriate residues for mutation. Thus, studying the 3D structures of AAV capsids enables us to further optimize and create efficient mutants to take them to the clinics for treatment of diseases like hemophilia.

#### Rational design of AAV variants via peptide insertion

Another approach to create novel AAV variant is to insert known ligands into the AAV capsid thereby allowing retargeting to specific cell types to which the WT vectors have a low affinity [51]. By this method it has been shown retargeting AAV vectors limits bio-distribution and improves specificity of transduction. For example targeted insertion of receptor-specific ligands or single-chain antibodies at the N-terminus of VP proteins has been tried out as early as 1998 by Yang *et al.,* [52] where the authors inserted a single-chain antibody against human CD34, a cell surface protein present on haematopoietic progenitor cells, at the 5′ ends of VP1, VP2 and VP3 resulting in an increased transduction of CD34-positive KG-1 cells. Later Wu *et al.,* [53] demonstrated that exchanging the HA epitope by the serpin receptor ligand KFNKPFVFLI78 resulted in a 15-fold higher infection of the lung epithelial cell line IB3 than by wild-type AAV-2. Targeting of rAAV-2 vectors by insertion of ligand coding sequences into the capsid genes was first done by Girod *et al.,* in 1999 [51]. In this study the authors inserted a 14 amino-acid peptide L14 (QAGTFALRGDNPQG) into the capsid DNA sequence. The L14 peptide contained a motif of the laminin fragment P1 which is the target for many integrin receptors that could be recognized by viruses for their cellular entry. The novel vector created by this peptide insertion could infect cell lines like B16F10 (mouse melanoma) and RN22 (rat swannoma) cell lines in contrast to wild-type AAV-2. AAV vectors are naturally hepatotrophic when injected systemically with varying propensity towards the liver [54]. However, it is sometimes desirable to get the AAV directed towards organs other than the liver. To this end Asokan *et al.,* [55] generated a hybrid vector AAV2i8 which contains a linear epitope of AAV8 on the heparin binding site of AAV2. In this study, using site-directed mutagenesis the authors replaced the hexapeptide motif 585-RGNRQA-590 (heparin sulfate footprint on AAV2 capsid) with corresponding amino acids from different AAV serotypes and non human primate isolates thereby generating a series of AAV2 inner loop (AAV2i) mutants. Amongst the several mutants that were created AAV2i8 displayed systemic biodistribution (more redirection to muscles) as compared to the wild type vectors when injected systemically in BALB/c mice. Additionally, the chimeric AAV2i8 also elucidated significantly less neutralization by anti-AAV2 serum or human serum. Because of its efficient retargeting to muscles, AAV2i8 can be a promising candidate for treating several musculoskeletal diseases. In a recent study, the authors created improved AAV vectors by rational engineering of capsid-glycan receptor interactions [56]. Two new vectors were created, AAV2G9 (dual glycan binding strain) and AAV2i8G9 (muscle tropic strain) by including the Gal binding footprint from AAV9 onto the VP3 backbone of AAV2 or the chimeric AAV2i8 with the help of structural aligning and site directed mutagenesis. The onset of gene expression from AAV2G9 (luciferase) was more rapid as compared to the parent vector AAV2 in Balb/c mice although the tropism was more or less towards liver (like AAV2). Further evaluation of the transduction profile of AAV2G9, revealed a significantly higher propensity towards heart (25 fold), kidney (4 fold), skeletal muscle (4 fold) and liver (4 fold) compared to WT-AAV2. The liver detargeted, muscle specific AAV2i8G9 also showed similar improvement in its transduction profile making it an ideal and optimal vector for systemic gene therapy of muscular dystrophies [57-59]. Bowles *et al.,* described a rationally designed chimeric AAV2.5 [59] used for a phase I clinical trial for Duchenne Muscular Dystrophy (DMD) in 2011. Following a rational approach, AAV2.5 was generated from the AAV2 capsid with five mutations from AAV1. The novel AAV2.5 vector not only had improved muscle transduction capacity like AAV1, but also had reduced antigenic cross reactivity against both AAV1 and AAV2. In this randomized trial with DMD boys (AAV vector injected into bicep muscle), no cellular immune response or any other adverse events were noticed against AAV2.5, although recombinant AAV genome were detected in all the patients establishing the safety and efficacy of the rationally designed AAV2.5 vector. Vandenberghe *et al.,* [60] described a hybrid vector AAV6.2 which contained a single F129L mutation in the phospholipase A2 domain. This vector demonstrated functional correction of cystic fibrosis transmembrane regulator in cultured human airway epithelia [61] as well as demonstrated efficient gene transfer compared to other AAV serotypes in mouse nasal airways and cultured human airway epithelia [62].

#### Directed evolution of AAV variants

Chimeric capsids based on *in vitro* evolution strategy was first described by Grimm *et al.,*, in 2008 [63]. In this study, cap sequences from 8 AAV serotyopes (AAV2,4,5,8,9, avian, caprine and bovine AAV) were randomly shuffled and reassembled and selected first for its ability to transduce hepatoma cell lines in the presence of IVIG. The authors were able to get a single mutant after the selection process, AAV-DJ, a chimera of AAV2, 8 and 9. AAV-DJ was shown to have a reduced host immune response compared to AAV8 and AAV9 at lower IVIG levels. Koerber *et al.,* in 2008 [64] created seven chimeric AAV vectors by shuffling capsid sequences of AAV1,2,4,5,6,8 and 9. One of the chimeric mutant vectors with a greater than 90% similarity to AAV1/6 showed a 400 fold more reduction to neutralization by IVIG compared to AAV2. Maheshri *et al.,* [65] utilized directed evolution approach to generate AAV with enhanced gene delivery capability. Using combinatorial library approaches the authors created two AAV2 derived mutants, AAV2.15 and AAV2.4 which contained mutations at critical antigenic sites. Both these vectors could resist neutralization from antibodies *in vivo* at serum levels which were much higher than what is required to neutralize the wild type vector. Additionally, both the mutants elucidated increased gene expression when compared to the wild type. Using DNA shuffling in an *in vitro* model of human ciliated airway epithelium, *Li et al.,* [66] were able to generate two AAV variants (harboring capsid components from AAV-1, AAV-6, and/or AAV-9) with improved efficiency (25% compared to the parental vectors) to deliver cystic fibrosis transmembrane conductance regulator (CFTR) gene to human ciliated airway epithelium isolated from cystic fibrosis patients. More recently Dalkara *et al.,* [67] successfully created a novel AAV vector to infect outer retina from the vitreous by utilizing the technique of directed evolution *invivo*. In this study they enriched for an AAV variant which showed widespread delivery to the outer retina and reverses the disease phenotypes of X-linked retinoschisis and Leber’s congenital amaurosis in murine models. This vector also efficiently transduced primate photoreceptors from the vitreous.

#### Peptide scanning for immunogenic epitopes

We have seen in clinical trials using AAV that in targeting immune competent sites like liver and muscles, preexisting humoral immunity acts as an impediment to long term gene expression. Thus it necessitates basic knowledge of immunogenic epitopes on AAV capsids to rationally design AAV variants that can evade this immune response. Peptide scanning to map neutralizing epitopes for antibodies against AAV capsid opens up another rational approach to bioengineer AAV capsids. In one such early study Moskalenko *et al.,* [68] identified 6 linear epitopes that are targets of neutralizing antibodies present in human serum samples. Mouse monoclonal antibody epitope was further identified by Wobus *et al.,* [69] where the authors mapped both linear and conformational immunogenic epitopes by using antibodies and peptide insertion mutants of AAV2 [51]. These AAV2 mutants displayed an integrin binding ligand, L14, at surface exposed positions of the capsid [69]. In later years further work was carried out by Huttner et al., [70] which led to the identification and validation of positions 534 and 573 on AAV2 capsid as the major antigenic determinants in humans. One important aspect to keep in mind is that altering the immune epitope on the AAV capsids should not change its transduction efficiency. To this end, Huttner et al., [70] created a mutant I-587 which was able to transduce B16F10 cells despite the presence of neutralizing antibodies. Further Perabo *et al.,* [71] and Huttner *et al.,* [70] demonstrated similar finding where insertion of peptides at 587 modulated both cell tropism and antibody neutralization.

## Conclusion

Although AAV has gained immense popularity as a gene therapy vehicle to treat several genetic disorders, there is still a persistent need to further improve on the vector capsid design and engineering which can bypass the problem of neutralization by preexisting antibodies as well as T-cell mediated immune clearance. Over the past decade, many technologies have been used to make the AAV capsids less immunogenic and more efficient. For example, coating of AAV particles by inert polymers like polyethylene glycol (PEG) has been shown to modestly decrease its immunological properties. Site directed mutagenesis of amino acid residues (S/T/K/Y) on AAV capsids based on their phosphorylation status and presence on B- cell epitope has created novel vectors with reduced antibody response as well as high transgene expression. Rationally creating point mutations does not seem to interfere with their overall safety profile or packaging efficiency when compared to wild type vectors. Thus, it enables us to achieve high gene expression at a low vector dose which will further reduce the chance of eliciting immune response against the viral particles. Also, inserting known peptides at specific sites on AAV capsids can alter the natural tropism of AAV which is extremely helpful for targeted gene delivery at specific organs. Finally directed evolution of AAV can create novel chimeric vectors which can also have reduced neutralizing antibody response along with high target site specificity.

An alternate strategy that can be employed in quest of a ‘stealth’ vector is to isolate/screen novel AAV’s from human sources [8]. This strategy can potentially minimize immune response against the viral capsids because the host immune surveillance will most likely treat them as self antigens. Overall, although considerable progress has been made in the field of capsid bioengineering, there is still a need to improve on the available tools and existing vectors along with continued search to find/design newer vectors which can be truly called as a ‘super vector’ that is independent of prexisting antibody and immune profile across different patients.

## Abbreviations

AAV: Adeno associated virus
ITR: Inverted terminal repeat
ORF: Open reading frame
LCA: Leber’s congenital amaurosis
LPL: Lipoprotein lipase
hF.IX: Human factor IX
NAb: Neutralizing antibodies
CD40: Cluster of differentiation 40
EGFR-PTK: Epidermal growth factor receptor protein tyrosine kinase
S: Serine
T: Threonine
K: Lysine
Y: Tyronie
A: Alanine
R: Arginine
F: Phenylalanine
L: Leucine
FK506: Binding protein-FKBP52
MHC: Major Histocompatibility class
DMD: Duchenne muscular dystrophy
PEG: Polyethylene glycol
CFTR: Cystic fibrosis transmembrane conductance regulator
